# Opinion of the Medical Press upon the Death under Nitrous Oxide

**Published:** 1889-12

**Authors:** 


Opinion of the Medical Press upon the Death under Nitrous-Oxide.—The *Lancet* says:—The record of so unusual an event as a fatality under this anæsthetic has, through the medium of the daily press, received a widespread circulation, and has produced much alarm and dread of the gas. Now that the facts are fully known, it becomes our duty to allay what at best are groundless fears, and to remove the slur which rests upon nitrous-oxide gas, as well as reiterate that we believe nitrous-oxide to be practically without danger if only patients, as well as administrators of the gas, would take a few simple precautions. The patient whose sad death we recorded in a preceding issue was 71 years of age, she was suffering from advanced fatty degeneration of the heart and was fully aware of her condition. Although she breakfasted at nine in the morning, and the teeth were not removed until noon, digestion appears to have been wholly suspended by the intense terror she experienced at the thought of submitting to the operation. She repeatedly told her daughter that she "knew she would not survive the operation." Added to the full stomach, and the extreme apprehension, both conditions liable to tell against even a healthy, much more a fatty heart, the patient wore very tight corsets, so tightly indeed was she laced that it was found difficult to remove the stays even by slitting up the laces with a knife. Although it would appear the dentist warned the patient to loosen whatever she had on which was tight, she merely loosened the neck wraps, leaving the more important corsets tightly laced. As soon as nitrous-oxide was commenced to be inhaled, the dentist noticed that respiration was insufficient, and urged deeper inspiration. Doubtless this weak breathing was due to the tightness of the clothing. When eventually the patient became unconscious, two teeth were removed and a hole drilled into the antrum, giving vent to pent-up secretion. Then the patient suddenly changed color, became livid, while respiration and heart action ceased, it is believed, simultaneously. During the subsequent performance of artificial respiration the undigested contents of the stomach were voided. There seems little doubt in this case that the impairment of respiration from tight lacing—distention of the stomach—told upon a heart itself badly diseased and weakened by the fear to which the patient had given way, and the most melancholy result followed. There can be no question that when any known degeneration of the heart muscle exists that the recumbent or semi-recumbent posture should be adopted, as can easily be accomplished in a dental chair, and corsets removed and all waistbands and strings undone. Where fear exists in the patient's mind, it is important to avoid a full stomach; a cup of beef-tea or a basin of bread-and-milk should form the meal preceding the operation. Reviewing all the circumstances of this most sad fatality, there is, we think, no reason for going back from the position which modern anæthetists have occupied, that nitrous-oxide is not only the safest anæsthetic we possess, but that when it is circumspectly given it is practically free from danger, save in cases of very advanced disease of the heart or lungs.

*The British Medical Journal* says:—Our Edinburgh correspondent last week drew attention to a death which took place in the operating room of a dentist after the administration of nitrous-oxide. The occurrence is fortunately very rare, but it seems advisable to ask whether this anæsthetic is as safe as it is usually considered to be. The patient, Lady Milne, was a woman aged 71, of stout build, and under treatment for fatty degeneration of the heart. She had been referred to the dentist by her medical man, in order to have two teeth removed and an opening made into the antrum. It appears that Lady Milne very much dreaded the operation, and was heard to remark she feared she would not survive it. At 9 A. M. she breakfasted, but it seems her food remained wholly undigested, for during the performance of artificial respiration, undertaken when breathing ceased, it was ejected unchanged. The operation took place at noon. The dentist noticed whilst he gave the gas that Lady Milne breathed very shallowly, and begged her to inspire more forcibly, but this she failed to do, for, as was afterwards discovered, her corsets and clothing were so tightly bound round her that normal breathing was simply impossible. As soon as consciousness had gone the teeth were extracted and the antrum perforated. It was then observed by the dentist and his assistant that Lady Milne had become livid, had ceased to breathe, and her heart had stopped beating. Although artificial respiration was resorted to, the tongue being drawn forward, while the stays were with difficulty slit up with a knife, and nitrate of amyl exhibited, she never showed signs of life. Medical aid was promptly summoned, but in vain.

That nitrous-oxide will be blamed is certain, but a dispassionate consideration of the circumstances seems to point rather to the neglect of precaution on the part of the patient than to any particular danger from the anæsthetic. It is clear that Lady Milne died from mechanical rather than from poisonous agency. Persons, even with fatty heart, although of course they are the least adapted for any kind of anæsthetic, take nitrous-oxide with impunity; but the recumbent posture, with absolutely loose clothing and a stomach not distended with gasses and undigested food, are conditions which can hardly be omitted without grave risk. The dentist who gave Lady Milne gas seems to have regarded her statement about her weak heart as merely the expression of a highly nervous woman, but we can not but think it is wise in all such cases to give the patient the benefit of the doubt, and adopt the precautions to which we have above alluded.

